# In *Drosophila melanogaster* the COM Locus Directs the Somatic Silencing of Two Retrotransposons through both Piwi-Dependent and -Independent Pathways

**DOI:** 10.1371/journal.pone.0001526

**Published:** 2008-02-06

**Authors:** Sophie Desset, Nicolas Buchon, Carine Meignin, Michael Coiffet, Chantal Vaury

**Affiliations:** 1 Centre National de la Recherche Scientifique (CNRS), UMR6247-GReD, Clermont Université; INSERM, Faculté de Médecine, BP38, Clermont-Ferrand, France; 2 Ecole Polytechnique Fédérale de Lausanne, Lausanne, Switzerland; 3 Department of Biochemistry, University of Oxford, Oxford, United Kingdom; Ecole Normale Supérieure de Lyon, France

## Abstract

**Background:**

In the Drosophila germ line, repeat-associated small interfering RNAs (rasiRNAs) ensure genomic stability by silencing endogenous transposable elements. This RNA silencing involves small RNAs of 26-30 nucleotides that are mainly produced from the antisense strand and function through the Piwi protein. Piwi belongs to the subclass of the Argonaute family of RNA interference effector proteins, which are expressed in the germline and in surrounding somatic tissues of the reproductive apparatus. In addition to this germ-line expression, Piwi has also been implicated in diverse functions in somatic cells.

**Principal Findings:**

Here, we show that two LTR retrotransposons from *Drosophila melanogaster*, *ZAM* and *Idefix,* are silenced by an RNA silencing pathway that has characteristics of the rasiRNA pathway and that specifically recognizes and destroys the sense-strand RNAs of the retrotransposons. This silencing depends on Piwi in the follicle cells surrounding the oocyte. Interestingly, this silencing is active in all the somatic tissues examined from embryos to adult flies. In these somatic cells, while the silencing still involves the strict recognition of sense-strand transcripts, it displays the marked difference of being independent of the Piwi protein. Finally, we present evidence that in all the tissues examined, the repression is controlled by the heterochromatic COM locus.

**Conclusion:**

Our data shed further light on the silencing mechanism that acts to target *Drosophila* LTR retrotransposons in somatic cells throughout fly development. They demonstrate that different RNA silencing pathways are involved in ovarian versus other somatic tissues, since Piwi is necessary for silencing in the former tissues but is dispensable in the latter. They further demonstrate that these pathways are controlled by the heterochromatic COM locus which ensures the overall protection of Drosophila against the detrimental effects of random retrotransposon mobilization.

## Introduction

Genome sequencing projects have revealed that eukaryotic genomes contain large numbers of repetitive sequences and mobile genetic elements. Retrotransposons, which are mobile genetic elements that move through an RNA intermediate in a process termed retrotransposition, are highly abundant and comprise nearly half of the human and a third of the Drosophila genome. It is thus essential that eukaryotic cells retain tight control over these potential invaders in order to protect their genomes from the mutational threat that they pose. This control is especially critical in the germline, where retroelement activity can create a mutational burden that is then transmitted to subsequent generations. As numerous metazoan elements display expression that is restricted to the reproductive apparatus, including the germ line and in surrounding somatic tissues, genomes have evolved specific mechanisms to protect these tissues by further restricting the expression of these elements.

It has become clear in the last few years that RNA interference (RNAi) plays a major role in ensuring this type of protection [1]–[3]. Three main RNA silencing pathways, acting at the post-transcriptional level and involving three distinct populations of small RNAs–siRNAs, miRNAs and piRNAs–have been reported to date. siRNAs (small interfering RNAs) are derived from processed double-stranded RNAs (dsRNAs) into siRNAs of 20-24 nucleotides (nt) in length. These siRNAs are loaded onto an RNA-induced silencing complex (RISC) as single-stranded siRNA molecules which then bind and cleave the target RNA [4]–[6]. miRNAs (microRNAs) of 22 nts in length are endonucleolytically processed from endogenous non-coding transcripts. After their production, they bind the miRISC to mediate RNA silencing. miRNAs are developmentally regulated and play an important role in gene silencing throughout development [7], [8]. Whereas siRNAs and miRNAs are derived from both the sense and antisense strands of their double-stranded precursors, piRNAs (for PIWI-interacting RNAs) are mainly derived from antisense strands and are produced from discrete genomic loci [9], [10]. piRNAs are from 26–30 nts in length and have been reported in germline cells of drosophila, mice, rats and humans [11], [12]. In mice and human, they are required for male fertility. In Drosophila, a subset of piRNAs, called rasiRNA for repeat associated small interfering RNAs, has been directly implicated in the protection of the fly germ line against selfish genetic elements such as retrotransposons and repetitive sequences [13]–[15].

*ZAM* and *Idefix* are two LTR retrotransposons that are generally silent in the genome of *Drosophila melanogaster* [16]. In the vast majority of lines, these elements do not start their replication cycle and no mobilisation is observed. These lines are denoted S (stable) lines. However, in certain lines, called unstable (U) lines, this control has been perturbed and both elements are highly expressed in the ovaries. As a result of this expression, multiple copies of *ZAM* or *Idefix* become integrated into the germ line and are transmitted through successive generations [17], [18]. Characterisation of the U line has shown that an active transposon-silencing process that targets *ZAM* and *Idefix* has been mutated in this line. The mutated locus has been identified and shown to be a heterochromatic locus called COM, which is located at position 20A2-3 on the X-chromosome [16]. Analysis of the replication cycles of *ZAM* and *Idefix* in U and S lines has indicated that *ZAM* transcripts are only present in the posterior follicular cells of the ovaries of U lines, and not in the ovaries of S lines. Similarly, *Idefix* transcripts are detected in the germarium of U line ovaries, but not in S lines. Overall, our previous work indicated that both elements are subjected to two types of control in Drosophila: first, a regulatory pathway silences the elements and prevents them from initiating their replication cycles. Second, if this silencing is lost, another control mechanism that relies on specific *cis*-regulatory sequences present in the elements themselves restricts their expression to specific somatic cells of the ovaries [19].

In this study, we have carried out an in-depth analysis of the intrinsic regulatory properties of *ZAM* and *Idefix*, examining the pathways that promote their silencing in the S lines. We show that the control of *ZAM* and *Idefix* is mediated by a homology-dependent *trans*-silencing pathway that displays characteristics of the rasiRNA pathway. At the same time, it displays three unique features: *i)* in addition to the reproductive apparatus, the silencing is exerted in most if not all of the somatic tissues of flies; *ii)* Piwi is required in cells of the reproductive apparatus, but it does not play a role in other tissues; *iii)* The function of COM is ubiquitous.

## Results

### The U5 region of the *ZAM* LTR is sufficient for the differential regulation between S and U lines

**Figure 1:**
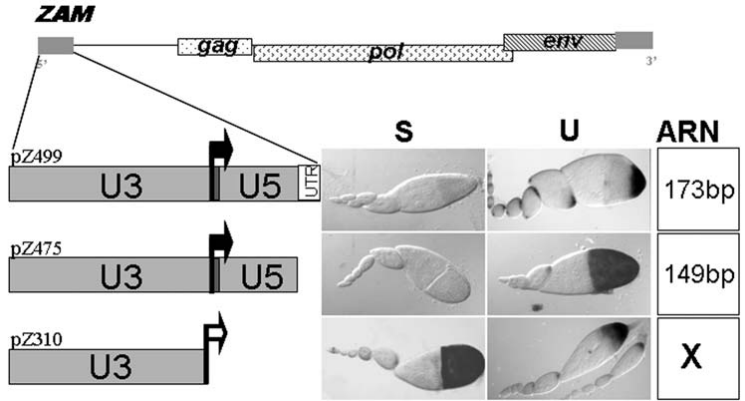
The U5, but not U3, region of the *ZAM* LTR is required for repression. The genomic structure of the *ZAM* retrotransposon is depicted at the top. Structures of the *lacZ* reporter trangenes used in this study are shown below on the left, and their expression in follicle cells from the S or U background are indicated at right. Transcripts initiated from the endogenous transcription initiation site of *ZAM* (black arrow) in transgenes pZ499 and pZ475 are homologous to *ZAM* over 173 and 149 bp, respectively. These transgenes are sensitive to the S or U status of the line as illustrated by the histochemical detection of β-galactosidase activity in the ovarioles. pZ310 contains the U3 sequence of the element and is expressed from a minimal heat shock promoter (white arrow) so that no sequence homologous to *ZAM* is present within the p310 transcript. Its expression is not under the control of the S or U status of the lines and is thus observed in the ovarioles from both the S and U backgrounds, as illustrated on the right.

We have previously reported that the transgene denoted pZ499, which contains the full-length LTR of *ZAM* and the first 26 bp of its 5′UTR (499 bp) fused to a *LacZ* reporter gene, responds to the two types of control over *ZAM* expression: i) repression, which depends on the fly genotype (U or S); and ii) tissue-specific activation, which drives expression in a very specific group of cells located at the posterior pole of the follicle [19]. The LTR is composed of a U3 region spanning nucleotides 1 to 325, a central R region from nucleotides 326 to 347, and a U5 region from nucleotides 348 to 473. The transcription initiation site defines the boundary between the U3 and R regions, and the polyadenylation site corresponds to the boundary between the R and U5 regions. To investigate the specificity of *ZAM* transcription in the different lines and to localise the sequences involved in its regulation, we analysed the expression of two additional transgenes placed in an S or U genetic background. These transgenes, pZ310 and pZ475, contain *ZAM* fragments extending from nucleotides 1 to 310 or 1 to 475, respectively, fused to the *LacZ* reporter gene. We found that pZ475 responds to both the strain- and tissue-specific controls that have been previously described for the full-length LTR [19]: its expression is restricted to the follicle cells of the U line and is absent in the S line (Fig. 1). By contrast, pZ310 which is expressed from a minimal heat shock promoter responds to the tissue-specific control that restricts its expression to the posterior follicle cells, but is insensitive to the line-specific control, since it is expressed in both the U and S genetic backgrounds (Fig. 1).

Therefore, we concluded based on this analysis of the pZ310, pZ475, and pZ499 lines that the line-specific expression of *ZAM* is controlled by sequences present in the R and/or U5 regions of its LTR.

### *ZAM* and *Idefix* are regulated by a homology-dependent gene-silencing mechanism

**Figure 2:**
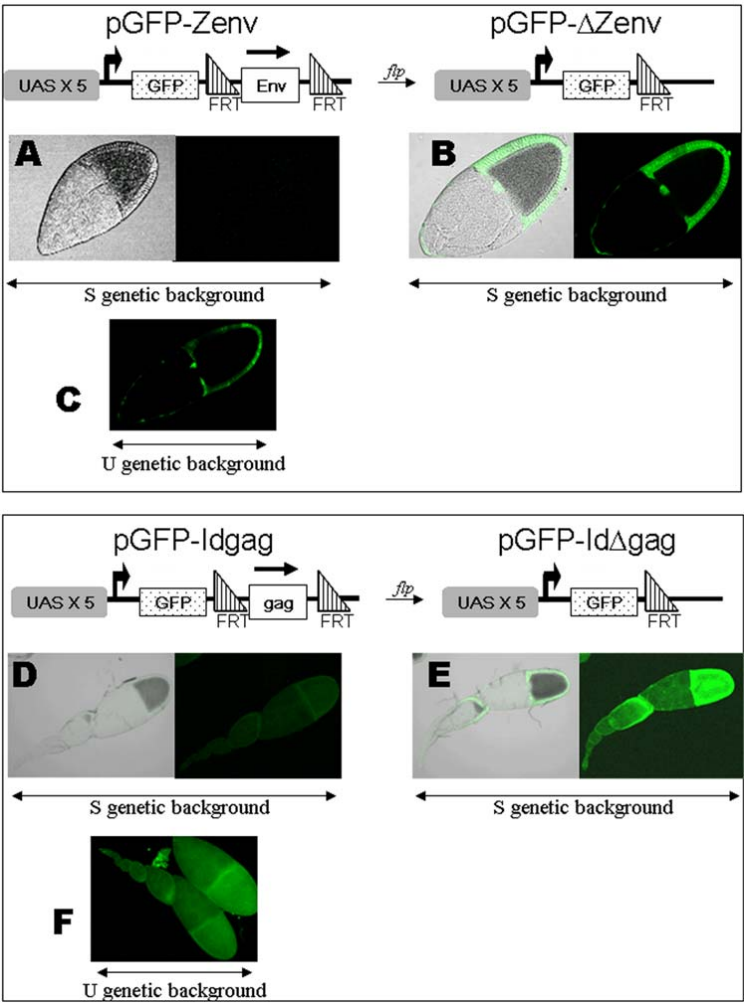
Transgenes with a GFP reporter gene fused to a *ZAM* sequence act as sensors of the repression. The genomic structures of the transgenes pGFP-Zenv and pGFP-Idgag used in this study are presented at the tops of both panels: The grey boxes correspond to the UASt promoter, the dotted boxes to the GFP gene, and the white box to the *env* fragment of *ZAM* or the *gag* fragment of *Idefix*. Triangles indicate the FRT sites. Focal plane of the follicles dissected from a line in which the pGFP-Zenv transgene is driven by the ubiquitous Actin-Gal4 driver. Expression of the pGFP-Zenv transgene in an S genetic background before (A) or after (B) *flp*-recombinase action, or in a U genetic background before the *flp* treatment (C). GFP expression in the ovarioles of a transgenic line bearing the pGFP-Idgag transgene driven by the ubiquitous Actin-Gal4 driver. Expression of the pGFP-Idgag transgene in an S genetic background before (D) or after (E) *flp*-recombinase action, or in a U genetic background before the *flp* treatment (F). No GFP is detected in ovaries of the S lines. Its expression is recovered after the flp treatment or when the COM locus is mutated, as in the U genetic background.

Transgenes pZ475 and pZ499 both initiate transcription from the *ZAM* promoter at nucleotide 326. When these transgenes are transcribed, 149 and 173 nucleotides, respectively, of *ZAM* are present at the 5′ end of the transcripts (Fig. 1, table). By contrast, the *ZAM* promoter is absent in pZ310, and no *ZAM* sequence is transcribed. To determine whether the *ZAM* promoter or the presence of a *ZAM* fragment within a chimeric transcript is responsible for the differential expression of *ZAM* in the S and U lines, we designed constructs in which a GFP reporter gene, driven by the UASt promoter, is fused downstream of its coding sequence and upstream of the polyadenylation site to diverse fragments of *ZAM* (Fig 2 A). Transgenic lines were established and tested for GFP expression in the ovaries. A 720 bp fragment from within the third ORF of *ZAM* was tested. This fragment corresponds to the region encoding the Env protein, spanning nucleotides 6385 to 7105 of the *ZAM* sequence. This *ZAM* fragment was inserted in an orientation such that transcription of the transgene would give rise to mRNA corresponding to the sense-strand fragment of *ZAM*. Furthermore, the *env* fragment was flanked by FRT elements, which are targets for *flp* recombinase. Transgenic lines were established and denoted pGFP-Zenv. The expression of the UASt transgenes was induced by crossing with flies containing a ubiquitous somatic actin-Gal4 driver in the S genetic background. Data obtained are presented in Fig. 2 A, B and C.

We examined the expression of pGFP-Zenv in the ovaries of three independent transgenic lines. The genotype of these lines was [S/S; actin-Gal4/CyO; pGFP-Zenv/pGFP-Zenv]. No fluorescence was detected in either of the lines (Fig. 2A). A number of these flies were subjected to *flp* recombinase action; to do this, they were crossed with flies expressing the *flp* recombinase under the control of a heat-shock promoter. Embryos laid by these females were then exposed to two heat-shocks at 38°C for 1 hr [20]. In these conditions, the efficiency of recombination between the two FRTs and the consequent deletion of the intervening *env* fragment is near 100%. The resulting flies were denoted pGFP-ZΔenv (Fig. 2B). Analysis of the expression of the pGFP-Zenv and pGFP-ZΔenv transgenes, activated by the actin-Gal4 driver in the ovaries, showed that whereas GFP is never expressed in the follicle cells when the pGFP-Zenv transgene is present in an S genetic background (Fig. 2A), fluorescence was clearly present in the same S background when the *ZAM* fragment was flipped out (pGFP-ZΔenv transgene Fig. 2B).

In a second series of tests, we compared GFP expression from the pGFP-Zenv construct in the S and U genetic backgrounds. We found that GFP is only expressed in U line flies, as shown in Fig 2C for a follicle with genotype [U/U; actin-Gal4/CyO; pGFP-Zenv/pGFP-Zenv]. It should be noted that because of the non-functionality of the UASt promoter in germinal cells, the absence of a signal in the nurse cells or the oocyte is not informative in these experiments.

When GFP expression was compared between the different transgenic lines using the same settings on the confocal microscope, we found that the GFP signals were always lower in the transgenic U lines bearing the pGFP-*ZAM* sensor transgene than they were with the flipped-out transgenes. This suggests that full expression of GFP might not be recovered in U lines, which may only partially release *ZAM* silencing. Alternatively, the pGFP-Zenv transcripts might be less stable than the shorter pGFP-ZΔenv transcripts. Although this latter hypothesis cannot be excluded, results obtained when analyzing expression of these transgenes through northern blot experiments favour the former one (see below).

In additional assays, 1 kb of the 5′ UTR of *ZAM* was placed downstream of the GFP gene, in the 5′ to 3′ orientation with respect to *ZAM* transcription. Similar tests as those performed with the pGFP-Zenv transgenic lines were performed. The regulation of the GFP gene in the so-called pGFP-ZU transgenic lines gave the same results as for pGFP-Zenv (data not shown).

We conclude that the silencing of the pGFP-Zenv and pGFP-ZU transgenes is under the control of a transposon silencing pathway that targets endogenous *ZAM* retrotransposons and that is absent in the U line.

As *Idefix* is also repressed in S lines and is active in U lines, we used P-element transformation to introduce additional constructs containing UASt repeats located upstream of the GFP reporter gene and different fragments of *Idefix* into the genome of S line flies. We tested two different fragments. One corresponded to a non-coding region from the 5′ UTR of the gene, and the second to a coding region taken from its *gag* gene. Similarly to the pGFPZU and pGFPZenv transgenes, the *Idefix* fragments were inserted in an orientation such that transcription of the transgenes would give rise to mRNA corresponding to the sense-strand fragment of *Idefix*. These constructs were respectively denoted pGFP-IdU and pGFP-Idgag. Expression of the GFP reporter gene under the control of the actin-Gal4 driver was assayed in the ovaries of flies that were homozygous for both the X chromosome of the stable S line and the pGFP-Id transgene. Results obtained when the *gag* gene from nucleotides 1003 to 1422 was placed downstream of GFP are presented in Figure 2 D, E, and F. Similar results were obtained with the pGFP-IdU lines (data not shown). No fluorescence was detected in the ovaries of this S line (Fig. 2D). This absence of fluorescence depends upon the S status of the line, because fluorescence was clearly observed when the stable X chromosome was replaced by an X chromosome from a U line (Fig. 2F). A *flp* recombinase assay was conducted on the transgenic S/S line, as depicted for pGFP-Zenv. When the *gag* fragment of *Idefix* was flipped out with the *flp* recombinase, giving rise to flies denoted pGFP-IdΔgag, fluorescence was clearly recovered in the ovaries of S line flies (Fig 2E). These assays were performed on four independent transgenic lines for each construct, giving the same results.

Thus, it appears that both *ZAM* and *Idefix* are controlled by a common silencing mechanism that has several definable properties. First, it is active in the follicle cells of S lines. Second, it does not function through a specific sequence present within both elements, but can rather target regions all along their lengths, from their 5′ to the 3′ ends. Finally, this silencing mechanism is disrupted in the U lines.

### Sense-strand transcripts of *ZAM* and *Idefix* are specifically targeted by the silencing machinery

**Figure 3:**
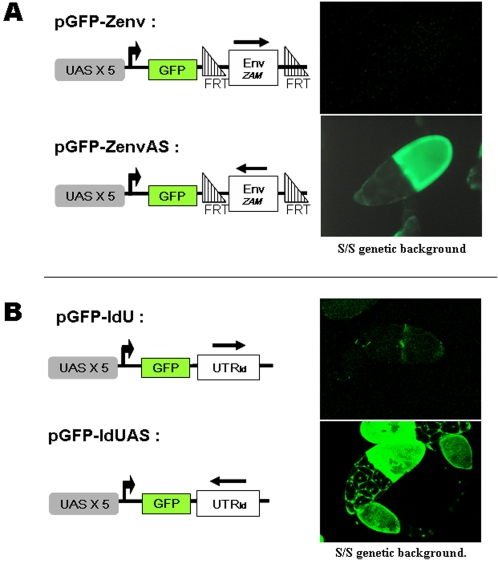
Transgenes bearing *ZAM* or *Idefix* sequences placed in an antisense orientation are not targeted by the repression. Expression of sensor transgenes carrying *ZAM* or *Idefix* fragments inserted in a sense and an antisense orientation. The genomic structure of the so-called pGFP-Zenv, pGFP-ZenvAS, pGFP-IdU, pGFP-IdUAS transgenes are depicted on the left. The orientation of the fragment is indicated by an arrow. The repression mechanism is able to discriminate between sense and antisense targeted sequences. In an S/S genetic background, only transgenes with ZAM and Idefix in an antisense orientation are correctly expressed. Clear fluorescence due to GFP expression is detected in the ovarian follicles of pGFP-ZenvAS and pGFP-IdUAS transgenes, as illustrated on the right.

A next set of experiments was performed with similar sensor GFP transgenes, but in which the fragments of *ZAM* and *Idefix* were inserted in the opposite orientation. Specifically, the 720 bp fragment within the third ORF of *ZAM* and the 456 bp fragment corresponding to the 5′ UTR of *Idefix* were tested in these experiments. These transgenes were denoted pGFP-ZenvAS and pGFP-IdUAS, respectively (Fig. 3). When transcribed, the resulting transgenes gave rise to transcripts which were antisense with respect to the endogenous *ZAM* or *Idefix* genomic RNAs. The ability of GFP to be expressed in the different lines was then assayed by introducing the actin-Gal4 transcription driver by crossing. While no fluorescence was observed when the expression of the (sense-strand) pGFP-Zenv or pGFPIdU transgenes was assayed, strong GFP fluorescence was detected in ovarian somatic tissues of the three independent (antisense) transgenic lines established with either pGFP-ZenvAS or pGFP-IdUAS (Fig 3 A and B). The intensity of the fluorescence was very similar to that observed with pGFP transgenes containing no *ZAM* or *Idefix* sequences, indicating that no silencing was exerted on these sensor transgenes.

Therefore, these transgenes are not sensitive to the repression exerted by the S genetic background. Further, this result suggests that the silencing mechanism that targets ZAM and Idefix is only directed against mRNAs containing sequences homologous to their sense-strand transcripts.

### Silencing of *ZAM* and *Idefix* occurs at the RNA level and involves small RNAs with characteristics of rasiRNAs

**Figure 4:**
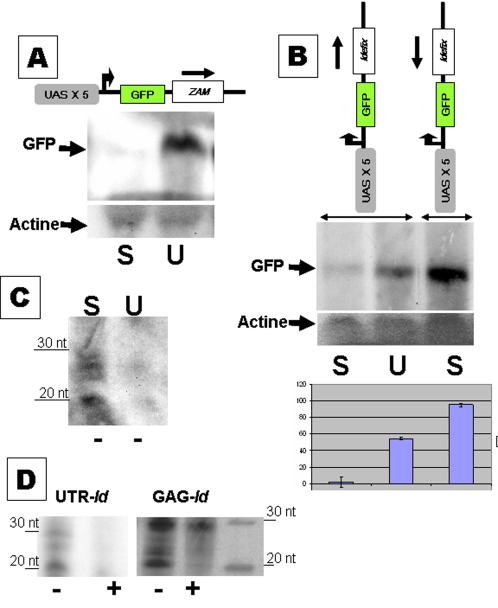
The repression machinery controlling *ZAM* and *Idefix* acts post-transcriptionally, before translation. A: Transcripts from the pGFP-ZU transgene were examined in northern blot experiments. A typical result is shown in A. GFP transcripts revealed by a riboprobe complementary to GFP mRNAs are detected in the U line and not in the S line. Actin is used as a loading control. B: Northern blots and quantification based on three northern blot experiments performed on flies containing pGFP-IdU and pGFP-IdUAS transgenes. Their structures are presented above the graph. No GFP transcripts synthesized from the pGFP-IdU transgene are detected by the GFP riboprobe in an S background, whereas their amount is high in a U background. An even higher amount of GFP transcripts is observed in an S or U background when the *Idefix* fragment is inserted in the opposite orientation (pGFP-IdUAS transgenes). C and D- RNase protection assays reveal the presence of small RNAs (20 to 30 nt long) that are homologous to *ZAM* and *Idefix.* These RNAs are detected in S lines and, at a much lower level, in the U line. Small RNAs homologous to the antisense strand of the 5′UTR of *ZAM* are presented in C. 20 to 30 nt long antisense strand RNAs (−) homologous to the 5′UTR or the *gag* gene of *Idefix* are detected. Sense strands (+) are absent or present in very small amounts. A typical experiment is presented in D. Signs (+) and (−) indicate respectively sense-strand and anti-sense strand RNAs of ZAM or Idefix revealed by the riboprobes.

The *trans*-silencing phenomenon described above was analysed through GFP expression of the transgenes. To determine whether this silencing acted at the translational level or at the RNA level, we looked for GFP RNA encoded by the transgenic lines. Total RNA was extracted from ovaries of transgenic lines in either the S or U genetic backgrounds, and northern blots were performed. The nylon filters were probed first with a riboprobe corresponding to the GFP gene and second to one corresponding to the actin gene as a loading control. Typical results are presented in Figure 4. GFP transcripts synthesized from the pGFP-ZU transgene were not detected in S lines, or only at a very low level, whereas they were abundant in U lines (Figure 4A). Similar results were obtained when transgenes bearing *Idefix* sequences were analysed (Fig. 4B). The quantitative analysis of the transcripts as observed on northern blots is also presented in Fig. 4B. Further, while no GFP RNA was detected in S lines in which the transgene encoded a transcript containing a sense-strand sequence of *Idefix,* GFP RNA was clearly detected in S lines in which the transgene included an antisense sequence of *Idefix* (Fig. 4B). In this context, the amount of GFP RNA was appreciably higher than that detected from transgenes with *Idefix* in the sense orientation in U flies.

These results indicate that the S line control does not act at the translational level but rather at the RNA level. As observed in the analysis of the sensor transgenes, the silencing is strictly directed against transcripts with sense-strand RNAs of *ZAM* or *Idefix*, and not against their antisense strands. Further, the silencing is released, although only partially, in the U genetic background.

The characteristics of this silencing were reminiscent of silencing involving rasiRNAs [13], [15], [21]. Therefore, we searched for putative small RNAs that are homologous to *ZAM* and *Idefix*. As northern blot experiments failed to identify any such small RNAs, we employed the more sensitive RNase protection assay. Even with this more sensitive assay, we could only barely detect small RNAs, indicating that they are very low in number. However, in view of the consistency of the results–obtained in at least three independent experiments performed with each sense and antisense probe from the 5′UTR, *gag*, *pol*, and *env* genes from both elements–certain general observations concerning the small RNA populations detected can be made as illustrated fig 4 C and D. First, short RNAs of 20 to 30 nt and homologous to *ZAM* and *Idefix* were detected, but no clear populations of any specific length can be defined at this stage of our detection assays (Fig. 4C and D). Second, these small RNAs were found in S lines as well as, although at lower levels, in U lines (Fig. 4C). Third, the abundance of short antisense RNAs was always higher than the almost undetectable short sense RNAs, suggesting that most of the detected small RNAs are antisense to *ZAM* and *Idefix* and single-stranded (Fig. 4D). Finally, small antisense RNAs were detected regardless of the probe used. Typical results obtained with the 5′UTR of *ZAM* (Fig. 4C) and with the 5′UTR or the *gag* gene of *Idefix* (Fig 4D) are presented.

These results show that the silencing machinery acting against *ZAM* and *Idefix* is associated with the presence of a small population of 20–30 nt RNAs, most of them being complementary to *ZAM* and *Idefix* mRNA.

### The silencing mechanism targeting *ZAM* and *Idefix* involves the PIWI Argonaute protein in the reproductive apparatus

**Figure 5:**
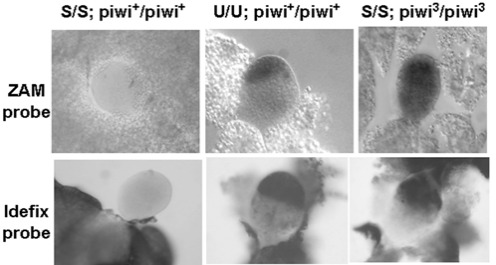
* ZAM* and *Idefix* are regulated by a PIWI-dependent pathway in the reproductive apparatus. *In situ* hybridization experiments reveal *ZAM* and *Idefix* expression in female gonads from third instar larvae. *ZAM* and *Idefix* transcripts are not detected in S flies with a wild-type *piwi* gene (left). As shown by the black staining, *ZAM* and *Idefix* mRNAs are detected in U flies with a wild-type *piwi* gene (middle). In S lines homozygous for the *piwi* ^3^ allele, *ZAM* or *Idefix* transcripts are no longer repressed, and their transcription is visualised in gonads (right). Probes used in these experiments are indicated on the left.

The Piwi protein has been shown to be involved in the rasiRNA pathway to maintain transposon silencing in the germline [1]. To test whether Piwi is also necessary for the repression of *ZAM* and *Idefix*, we first investigated the effect of the *piwi* ^3^ mutation on the expression of endogenous *ZAM* and *Idefix* elements in the ovaries of S-line flies (Fig. 5). Because the morphology of homozygous *piwi* ^3^ ovaries is severely affected in adult flies, it was impossible to investigate *ZAM* and *Idefix* expression in adult ovaries. We thus performed experiments in the gonads of third instar larvae. By *in situ* RNA analysis using strand-specific riboprobes for *ZAM* and *Idefix*, we found that both of the elements are expressed in female gonads of third instar larvae from the U line, as shown in a homozygous [U/U; *piwi* ^+/+^] genetic background (Figure 5, middle). No staining corresponding to *ZAM* or *Idefix* RNA was ever detected in the gonads of larvae having the corresponding genotypes in the S line [S/S; *piwi* ^+/3^] (not shown) or [S/S; *piwi* ^+/+^] (Figure 5, left). By contrast, clear expression of *ZAM* and *Idefix* was observed in the homozygous genetic background [S/S; *piwi* ^3/3^], displaying a pattern of expression similar to that detected in the U line (Fig. 5, right).

These findings provide evidence that Piwi is a component of the pathway that silences *ZAM* and *Idefix* in the ovarian somatic tissue.

### The silencing mechanism controlling *ZAM* and *Idefix* is active in somatic tissues throughout fly development

We next investigated whether the silencing mechanism involved in the repression of *ZAM* and *Idefix* is strictly restricted to follicular cells, where proper *ZAM* and *Idefix* enhancers are active, or if it is more widely present and active in other tissues. To address this question, the expression of the pGFP-*ZAM* and pGFP-*Idefix* transgenes was examined throughout fly development, in embryos, larvae, and adult flies. Two Gal4 drivers were used in these experiments: the ubiquitous actin-Gal4 driver, as described above, and the 24B-Gal4 driver, which is specifically expressed in mesodermal cells [22].

**Figure 6:**
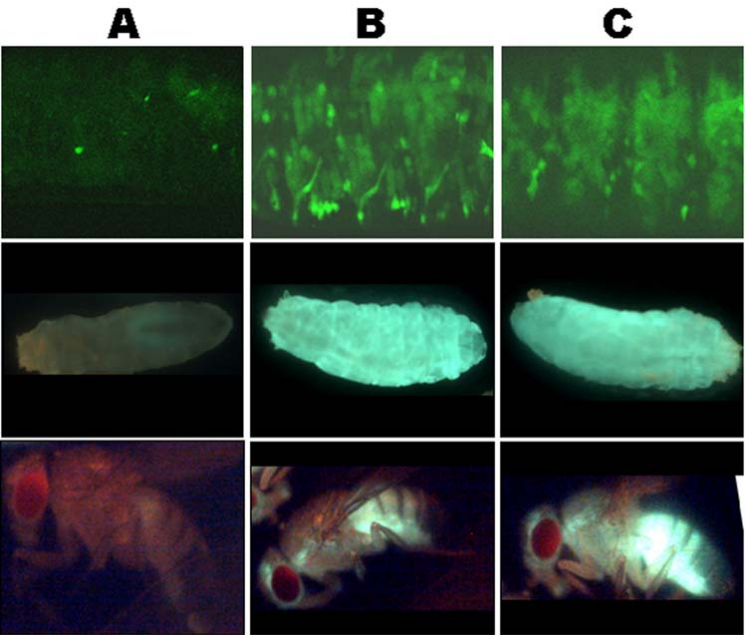
The silencing mechanism targeting ZAM and Idefix is active in somatic tissues throughout fly development. A) In an S/S genetic background, the pGFP-IdU sensor transgene driven by 24B-Gal4 is not expressed in embryos, larvae, or adults (top, middle and bottom panels, respectively). Only a very faint level of fluorescence, corresponding to the background expression of GFP, is detected. B) In a U/U genetic background, the GFP-IdU transgene silencing is released and GFP fluorescence is clearly observed in the three stages analyzed. The fluorescence pattern recapitulates the expression of the HOW gene in muscle and tendon cells, as expected for the 24B-Gal4 driver [22]. C) In an S/S genetic background, the pGFP-IdUAS sensor transgene carrying the 5′UTR of *Idefix* in the opposite orientation is not subjected to the silencing exerted on the *Idefix* sequences. pGFP-IdUAS is correctly expressed and GFP is detected in embryos, larvae, and adult flies.

In the S/S genetic background, no fluorescence was detected with any of the transgenes (pGFP-ZU, pGFP-Zenv, pGFP-IdU or pGFP-IdGag), regardless of the driver used (actin-Gal4 or 24B-Gal4). It should be noted that, if the microscope settings are optimized, a very faint level of fluorescence can be detected at each stage of development. This transgene silencing was observed in all the cells examined and throughout fly development, including in embryos, larvae, and adult flies. As an example, results obtained with pGFP-IdU driven by 24B-Gal4 are presented Fig. 6, column A. In contrast, when the X-chromosome in S-transgenic lines was replaced by one from a U line, clear fluorescence resulting from the expression of the GFP reporter gene driven by 24B-Gal4 was detected in embryos, larvae, and adult flies (Fig. 6 column B).

To determine whether the silencing mechanism that is active in these somatic tissues specifically targets sense-strand RNAs of *ZAM* and *Idefix*, similar experiments were conducted using the pGFP-ZenvAS and pGFP-IdUAS transgenes described in Fig. 3. We found that no silencing occurred on these transgenes, with both giving rise to clear ubiquitous GFP expression in all the examined stages. Results obtained with pGFP-IdUAS are shown in Fig. 6, line C.

It thus appears that the repression machinery targeting ZAM and Idefix is not restricted to ovarian follicle cells of S lines but is instead active in a broad range of cells (if not all) throughout fly development. This machinery is able to discriminate between sense- and anti-sense strand transcripts of ZAM and Idefix, and is under the control of the COM locus.

### The silencing mechanism active in somatic cells does not involve the PIWI Argonaute protein

**Figure 7:**
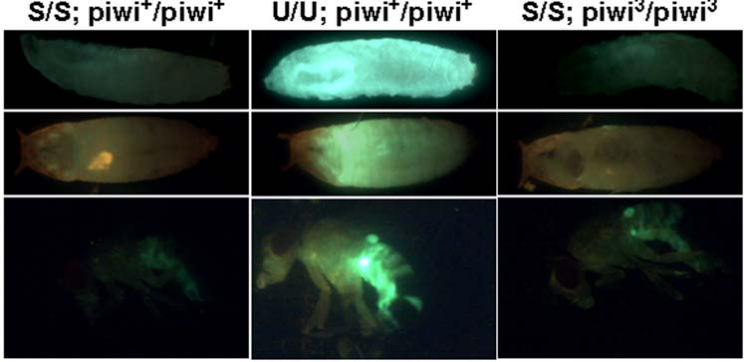
* ZAM* and *Idefix* are regulated by a PIWI-independent pathway outside of the reproductive apparatus. The pGFP-ZU sensor transgene driven by 24B-Gal4 is not expressed in larvae, pupae, or adult stages in a [S/S; piwi+/+] genetic background (right panel). Only a very faint level of fluorescence, corresponding to the background, is detected. A clear GFP expression is observed in these stages of development in a [U/U; piwi+/+] line (middle panel). In *piwi* mutant backgrounds, in homozygous [S/S; piwi3/3] lines, the silencing of the sensor transgene is not released. A very faint fluorescence level similar to that observed in homozygous [S/S; piwi+/+] lines is observed (left panel).

Since the control exerted by COM is active in the somatic cells outside of the reproductive apparatus, the question arose then to know whether Piwi is also a component of this somatic silencing pathway. Indeed, in addition to its function in the reproductive apparatus of flies, diverse functions have been attributed to Piwi in somatic tissues at different stages of fly development [23]–[26]. Thus, GFP expression of the sensor transgenes pGFP-*ZAM* or pGFP-Id, driven by 24B-Gal4, was analyzed in larvae, pupae, and adult S flies mutated or not for the *piwi* gene. *Piwi* ^2/2^, *piwi* ^3/3^, and transheterozygous *piwi* ^3/2^ mutations were tested in these experiments. The results obtained indicated that the silencing of the sensor transgenes in the somatic tissues of the fly outside of the ovaries does not depend on the presence of the Piwi protein. Indeed, in contrast to what was observed in the gonads, GFP expression was not recovered in the somatic tissues of S lines when the Piwi gene was mutated (see Figure 7 and data not shown).

These findings provide evidence that Piwi is not required for the silencing pathway present in somatic tissues outside of the reproductive apparatus.

## Discussion

### The silencing machinery targeting *ZAM* and *Idefix* involves the rasiRNA pathway

The rasiRNA pathway has been implicated in the silencing of several repeated genetic elements in the drosophila genome, such as *roo*, *Ste,* and the LTR retrotransposon *gypsy* [13], [27]. In this study, we have shown that the rasiRNA pathway, which is based on the strict recognition of sense-strand RNAs, is likely to be involved in the silencing of two additional LTR retrotransposons from *Drosophila melanogaster*, *ZAM* and *Idefix*. rasiRNAs have been reported to arise mainly from the antisense strand of retrotransposons or repetitive sequences [15]. Consistent with this, a profound strand bias for the silencing of the+strand of both *ZAM* and *Idefix* was observed. Further, rasiRNAs have been reported to consist of single-stranded RNAs of 25-30 bases in length, and in our experiments we detected 20-30 nucleotide long small RNAs corresponding to antisense strands of *ZAM* and *Idefix*. Most of these RNAs also appear to be single stranded, since most of them were only detected using a probe complementary to antisense strand *ZAM* and *Idefix* RNAs. The 30-nucleotide antisense strand RNAs might be effectors of a rasiRNA pathway that silences *ZAM* and *Idefix*. At the same time, the detection of RNAs of 20 bases in length is consistent with the possibility that an additional pathway could contribute to the establishment of complete silencing.

The silencing of *ZAM* and *Idefix* is not only ensured in the ovaries as already described for other transposable elements, but our data further provide evidence that it is also ensured in somatic tissues of the whole fly from embryos to adults

In *Drosophila* cells, a surveillance machinery is thus capable of specifically detecting genomic mRNA from both of these retrotransposons and interpreting their synthesis as an ongoing invasion that has to be countered because it would be ultimately harmful.

Transposable elements are generally viewed as genomic forces that are able to contribute to genomic diversity [28]. With this in mind, it is interesting that the expression of genes which have been subjected to integration of *ZAM* or *Idefix* in the antisense orientation will not be affected by the silencing machinery. The strand bias can thus preserve this source of genetic innovation brought by these nucleic invaders.

### The COM locus directs silencing through either a Piwi-dependent or -independent silencing pathway in different tissues

To ensure the silencing of endogenous genetic elements in the germline or in surrounding somatic cell lineages, rasiRNAs function through Piwi, a protein of the *Drosophila* Argonaute family [1], [3], [13]–[15]. Consistent with data reporting the tissue-specific expression of Piwi in ovaries [14], [29], our data indicate that Piwi is required for the silencing of *ZAM* and *Idefix* in ovarian tissues. However, several data have reported that Piwi is also active outside of the reproductive apparatus. It has been implicated in the regulation of transgenes expressed in tissues where the Adh gene is expressed [24], in the salivary glands [30] or recently in the eyes [26]. Furthermore, Piwi has been found to colocalize with PcG bodies, and its was suggested that it could regulate the nuclear organization of PcG chromatin targets [23]. Despite these somatic functions of Piwi, our data indicate that the somatic silencing of *ZAM* and *Idefix* which is also based on the strict recognition of their sense-strand RNAs, is Piwi-independent.

Our results further demonstrate that in the whole fly, the silencing of *ZAM* and *Idefix* is under the control of the *COM* locus [16], [31]. This discrete genomic locus, *COM*, is located at the heterochromatic 20A locus of the Drosophila X-chromosome and suppresses the activities of *ZAM* and *Idefix* [16], [31]. It displays several specific characteristics. It is located in the same region as the *flamenco* gene which controls the mobilisation of the retrotransposon *Gypsy* [31]. Further, it is mainly composed of defective transposon copies (BDGP release 5). Recently, it has been shown to be a piRNA cluster, and piRNAs homologous to defective copies of *ZAM*, *Idefix,* and *Gypsy* emitted by this locus have been reported [9]. Brennecke et al further reported that a link exists between *flamenco*-derived piRNAs and *gypsy* suppression. Based on their results, they proposed an amplification loop, the “ping-pong” model, to account for piRNA biogenesis. According to this model, sense transcripts from transposons are cleaved by Piwi or Aub RISC loaded with a piRNA guide. The cleaved transcript is not merely degraded but is also used to program Ago3 RISC. This complex, in turn, cleaves the antisense transcripts that originate from master loci such as the 20A locus. Again, the cleaved RNA serves to program Piwi or Aub RISC. Thus sense and antisense transcripts fuel an amplification cycle. This scenario is consistent with most of the characteristics of the silencing mechanism that targets *ZAM* and *Idefix* in the ovaries. However, if a link exists between the *COM*-derived small RNA and the silencing of *ZAM* and *Idefix*, it should implicate another protein than Piwi in the soma. Furthermore, an important piece of data originating in our study remains obscure. Although our genetic analysis points to *COM* as a master regulator of *ZAM* and *Idefix* silencing, some sequences from endogenous *ZAM* are absent from the *COM* locus (release 5 and Hadi Quesneville personal communication). For example, none of them is complementary to the 5′UTR fragment of *ZAM,* whose sequence has been directly implicated in the silencing of the pGFP-ZU transgene. A direct interaction between rasiRNAs derived from the 20A locus and these targeted transgenes seems thus to be excluded. In the ping-pong model, the need for mutual complementarity keeps the production confined to one pair of complementary piRNAs, preventing piRNA generation from spreading along a primary transcript as allowed by RNA-dependent RNA polymerase (RdRP)-mediated amplification. If piRNA emitted from the COM locus cannot spread along primary *ZAM* or *Idefix* transcripts, then an additional step in the piRNA mechanism should exist to ultimately direct destruction of transcripts bearing any fragment of *ZAM* or *Idefix*. More data are then necessary to understand how such piRNA are generated and what their exact role is in the control. Our present data, however, already implicate the ubiquitous activity of COM coupled to different factors in various tissues.

## Materials and Methods

### *Drosophila* strains

The S line *w^1118^* and the U line Rev were from the collection of the Institut National de la Santé et de la Recherche médicale UMR384. The ubiquitous actin-GAL4 and the mesodermic 24B-Gal4 drivers used are both located on chromosome 3. The 24B-Gal4 driver was a gift from K. Jagla.

All transgenic lines were obtained by injection of indicated transformation vectors into *w^1118^*. All stocks were maintained at 20°C. Expression of transgenes in a genetic context allowing *ZAM* and *Idefix* mobilisation was obtained in the progeny of crosses performed with transgenic lines (S genotype) and the U line described in [32]. Flies used for analysis of expression were raised and kept at 25°C.

### Transgenic constructs

pZ499 is the p*ZAM* construct described in [19]. It contains a *ZAM* LTR upstream of the LacZ reporter gene. In pZ475 and pZ310, the *ZAM* LTR is shortened at its 3′ end, resulting in fragments encompassing nucleotides 1 to 475 and 1 to 310, respectively, with respect to the *ZAM* sequence. pZ310 contains a minimal hsp70 promoter between the *ZAM* fragment (corresponding to the U3 part) and the LacZ gene. The pUASt-GFP vector was used for sensor transgene experiments. For pGFP-ZU and pGFP-IdU, the *ZAM* UTR (from nucleotide 475 to 1841) and *Idefix* UTR (from nucleotide 502 to 1024) were cloned downstream of GFP. pGFP-Zenv includes 720 bp of the *ZAM env* coding region (6385–7105), and pGFP-Idgag includes 1419 bp homologous to the *Idefix gag* coding region (1003–1422). In these transgenic constructs, the *ZAM* or *Idefix* fragments were cloned to be transcribed in the sense orientation, i.e. in a 5′ to 3′ orientation, with respect to endogenous *ZAM* and *Idefix* transcription. In pGFPZenvAS and GFPIdUAS, the *ZAM* or *Idefix* fragments were cloned in the opposite orientation.

### Histochemical staining for β-galactosidase

Ovaries were dissected in 1× phosphate-buffered saline (PBS), fixed in 0.5% glutaraldehyde in PBS for 5–10 min at 4°C, and rinsed twice in 1× PBS and once in Fe/NaP buffer [0.003 M Na_2_HPO_4_, 0.072 M NaH_2_PO_4_, 0.003 M K_3_Fe(CN)_6_, 0.003 M K_4_Fe(CN)_6_ 0.15 M NaCl, 0.001M MgCl_2_]. Staining was performed in Fe/NaP buffer with X-Gal (0.2 mg/ml final concentration) at 37°C. All samples were stained simultaneously and for the same length of time (2 hrs). Stained tissues were washed four times in 1× PBS, mounted in 1∶1 PBS:glycerol, and examined under an Axiophot microscope (Zeiss) using Nomarski optics.

### Fluorescent microscopy

Light and fluorescence microscopy was performed with an Olympus confocal microscope or an Olympus SZX12 binocular and a CCD color view camera. Comparisons between stable and unstable lines were carried out using the same acquisition settings.

### Flip-out experiments

The *hs*FLP flies (*w^1118^, hsp-FLP*; *cu kar2 Sb*/TM6, *Ubx e^5^*), kindly provided by Kent Golic, express *flp* recombinase under the heat shock promoter hsp70. Virgin *hs*FLP females were crossed with transgenic males for 24 hrs on cornmeal-glucose-yeast media at 20°C. Heat shocks of embryos <24 hrs old were performed as described by Ahmad and Golic (1996).

### Northern blot experiments

Total RNA from adult flies was extracted by Trizol, and 40 µg of total RNA was resolved on 1% denaturing agarose gels and probed with radiolabelled transcribed probes homologous to GFP. An actin 5C probe was used as a loading control. Experiments were repeated three times. GFP signals were quantified with a Biorad S125 phosphorimager.

### RNase protection assays

Small RNAs from adult flies were extracted using the Ambion *mir*Vana™ miRNA isolation kit. Aliquots of 5 µg of small RNAs were used in RPA experiments. Radiolabelled RNA probes homologous to the 5′UTR regions of *ZAM* or *Idefix*, or to the *gag* gene of *Idefix,* were 400 to 500 bases long (ZAM UTR from nucleotide 1027 to 1515, Idefix UTR from nucleotide 567 to 1010, Idefix gag from nucleotide 1028 to 1422). 5×10^4^ cpm of specific activity probe was used. As indicated for the Ambion *mir*Vana™ miRNA detection kit, hybridization was performed overnight at 42°C, and protected fragments were digested for 45 minutes at 37°C by RNase A/RNase T1. After RNase inactivation, protected fragments were precipitated and separated on a 15% acrylamide/polyacrylamide (19:1) gel running in 0.5×TBE. Protected fragments were detected by autoradiography.

### *In situ* hybridization

*ZAM* and *Idefix* mRNA expression was detected by *in situ* hybridization using DIG-labelled RNA probes transcribed from the pBS plasmids containing *ZAM* (3830 to 8040) or *Idefix* (4866–7191) fragments, using the kit from Roche. Ovaries from third instar larvae were dissected in phosphate buffer saline (PBS). Dissected ovaries were fixed in 5% formaldehyde for 20 min. Ovaries were rinsed with PBT (PBS, 0.1% Tween 20) prior to proteinase K treatment. Hybridization was performed in hybridization solution (50% formamide, 5× SSC, 0.1% Tween 20, 50 µg/ml heparin, 100 µg/ml salmon sperm DNA, and 100 µg/ml yeast tRNA) at 45°C overnight and was followed by washes in a 1∶1 mixture of hybridization solution and PBT at 45°C for 30 min each, and in PBT at room temperature (two washes of 20 min each). The hybridized probe was detected using the DIG nucleic acid detection kit (Roche).
